# Red Cross Medical Bureaus

**Published:** 1918-03

**Authors:** 


### The Medical Bulletin

N° 5 PARIS MARCH 1918

### RED CROSS MEDICAL BUREAUS

The following classification of the Medical Division of the American Red Cross is here presented to avoid confusion and to assist those who apply for information, direction, or help.

*Department of the Medical Advisor of the American Red Cross.*

Head of Division, Major Alexander LAMBERT, M. R. C., Medical Advisor of the Commission of the American Red Cross, 4, Place de la Concorde.
This Department controls the scientific research and publication activities of the medical branch of the American Red Cross.

*The Medical Bulletin.* Edited by Mr. WATSON, 6, rue Piccini.

For the publication of current medical information to be circulated among the doctors of the American Red Cross and of the Allied Forces.

*Medical Library.* Librarian, Mr. ESTES, 12, Place Vendôme.

For the use of doctors wishing to do research work or to use the medical reference library. A stenographer is employed for the convenience of doctors using the library. It is hoped that the library may become a meeting place for doctors and a center for information on the medical work of the American Red Cross and of the Army.

*Bureau of Medical Information.*

It is intended to build up a service for the collection of medical information of every kind.

*Research Committee of the American Red Cross.*

This Committee controls all medical and surgical investigations supported by the Research Fund of the American Red Cross. One activity of this Committee is the direction of the monthly meetings of the Research Society. Secretary of the Committee, Major Kenneth TAYLOR, M. R. C., 6, rue Piccini.

*Red Cross Research Society.* Monthly Meetings.

These meetings aim to bring the American medical personnel into direct touch with the representatives of the Allied Armies who attend them, and to give our men the benefit of a large experience in actual war work. The Society also affords them an opportunity to present new work to an international body of physicians. Notices of dates, places of meeting, etc., are sent out by the Secretary of the Research Committee.

*Medical Division, Department of Military Affairs.*

Head of Division, Lieutenant C. C. Burlingame, M. R. C.

To this department should be referred all matters dealing with the business side of the medical work of the American Red Cross connected with the following services :

American E. F. Hospitals.
Medical Service for American E. F. Auxiliaries.
A. R. C. Military Hospitals.
A. R. C. Convalescent Homes and Hospitals.
Medical Service for Civil Population and Dispensaries.
Nursing Service (Military Affairs).
Diet Kitchens.
Artificial Limb Service.

*Women's Bureau of Hospital Service.*

Head of Bureau, Miss Ruth Morgan.

This bureau has charge of the enrollment, equipment, assignment, care during illness and convalescence, vacations, and all other matters pertaining to trained nurses and to nurses' aids.

Chief Nurse, A. R. C., Miss Julia Stimson.

